# The Sustainable Potential of Single‐Ion Conducting Polymers

**DOI:** 10.1002/cssc.202500055

**Published:** 2025-05-02

**Authors:** Freddie J. Leslie, Kieran G. Stakem, Georgina L. Gregory

**Affiliations:** ^1^ Chemistry Research Lab University of Oxford 12 Mansfield Road Oxford OX1 3TA UK

**Keywords:** batteries, energy storage, ionic monomers, polycarbonates, single‐ion conducting polymers

## Abstract

Energy storage technologies are critical for sustainable development, with electrolyte materials playing a decisive role in performance and safety. Single‐ion conducting polymers (SICPs) represent a distinct materials class characterized by selective ion transport through immobilized ionic groups. While their potential for battery applications is recognized, an analysis of their sustainability implications and pathways to practical implementation has been lacking. This work demonstrates how strategic design of SICPs can contribute to sustainable energy storage through both materials’ development and device integration. Recent advances in lithium borate‐based systems and CO_2_‐derived polycarbonate architectures have achieved ionic conductivities exceeding 10^−4^ S cm^−1^ at room temperature through scalable synthesis routes. In lithium–metal batteries, their high transference numbers and viscoelastic properties enable stable cycling with industrial‐relevant cathode loadings, while as electrode binders, they enable aqueous processing and enhanced interfacial stability. Their versatility extends to sustainable chemistries, including sodium and zinc systems. Analysis reveals that while SICPs can enhance energy storage sustainability through improved performance, processability, and potential recyclability, opportunities remain in investigating end‐of‐life management. This work highlights frameworks for advancing SICP sustainability while maintaining the performance requirements for practical implementation in next‐generation energy storage.

## Graphical abstract

Single‐ion conducting polymers (SICPs) advance sustainable energy storage by combining CO_2_‐derived polycarbonate synthesis with efficient thiol‐ene and networking strategies for ionic functionality. Their selective lithium‐ion transport enables applications as electrolytes, binders, and protective interlayers in next‐generation batteries. Dynamic recyclability and scalable production underscore their potential for sustainable energy technologies.

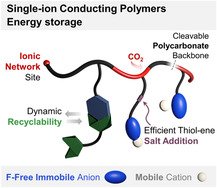

## 1 Introduction

Transitioning toward sustainable energy systems has become one of the defining challenges of the 21st century.^[^ ^1^ ^]^ Solutions must simultaneously address the challenges of environmental pollution, energy security, and climate change through a diverse range of technologies, including batteries,^[^ ^2^ ^]^ photovoltaics,^[^ ^3^ ^]^ fuel cells,^[^ ^4^ ^]^ and supercapacitors.^[^ ^5^ ^]^ Polymeric materials have proven instrumental in advancing these technologies, offering unique advantages, including mechanical flexibility, processability, and tunable properties.^[^ ^2^, ^3^, ^4^, ^5^ ^]^ Their versatility has enabled breakthrough innovations across next‐generation lithium and flow batteries to advanced photovoltaic systems and fuel cells.^[^ ^6^ ^]^ However, the widespread deployment of polymer‐based energy technologies necessitates careful consideration of sustainability across their entire lifecycle, from synthesis and processing to performance enhancement and end‐of‐life management.^[^ ^7^ ^]^ Developing environmentally conscious synthetic strategies and high‐performance polymers for energy applications represents a key direction in materials chemistry and sustainable technology.

**Figure 1:**
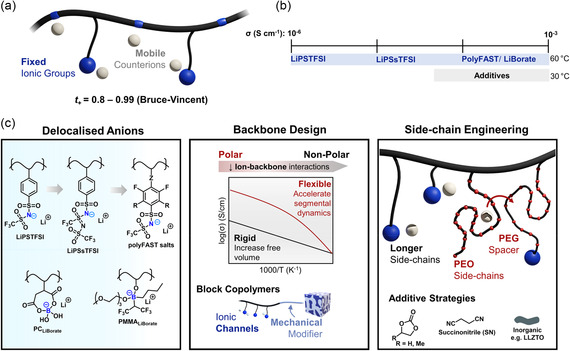
a) Schematic representation of SICP architectures showing anionic groups (blue) in pendant (more common) and backbone‐integrated positions (e.g., ref. [69]). This fundamental design enables sustainable materials development opportunities evaluated in this work. b,c) Current state‐of‐knowledge for enhanced SICP Li‐ion conductivity (*σ*), compiled from recent reviews[^9^, ^70^] and developments in delocalized anions,^[^ ^13^, ^37^ ^]^ backbone chemistry,^[^ ^16^, ^17^ ^]^ nanostructured block copolymers,^[^ ^18^ ^]^ side‐chain engineering,^[19b]^ additives^[^ ^20^ ^]^ and composites.^[^ ^28^ ^]^

Single‐ion conducting polymers (SICPs) are a promising materials platform for advancing multiple energy technologies and have shown particular advantages in rechargeable batteries.^[^ ^8^ ^]^ Unlike traditional dual‐ion conducting polymers where both cations and anions are mobile, SICPs feature one ionic species immobilized through attachment to the polymer backbone, leaving the counter‐ion as the sole mobile charge carrier (**Figure** **1**a). Typically, and for the focus of this work, this is a fixed anion with free Li‐cation. This design enables near‐unity transference numbers (*t*_+_ > 0.8) and suppresses detrimental concentration gradients, mimicking the selective ion transport found in inorganic and biological systems.^[^ ^9^ ^]^ These properties make SICPs particularly promising for energy applications, from longer‐life, dendrite‐resistant lithium metal batteries^[^ ^10^ ^]^ to faster response, lower power consumption actuators for biometric devices,^[^ ^11^ ^]^ and efficient membrane technologies.^[^ ^12^ ^]^

Recent advances have significantly improved the previously limiting ionic conductivities of these materials, particularly for Li‐ion transport (Figure 1b,c).^[9b]^ These improvements stem from multiple synergistic strategies: promoting ion dissociation via delocalized anions,^[^ ^13^ ^]^ creating dedicated ion channels,^[^ ^14^ ^]^ and preventing ion aggregation while accelerating polymer segmental dynamics.^[^ ^15^ ^]^ Such results have been achieved through modified backbone polarity,^[^ ^16^ ^]^ copolymer formation^[^ ^17^ ^]^‐particularly block architectures,^[^ ^18^ ^]^ side‐chain engineering,^[^ ^19^ ^]^ and additives.^[^ ^20^ ^]^ However, as SICPs approach practicality, their sustainability implications remain largely unexplored. Critical questions persist regarding scalable synthetic pathways, renewable feedstock integration, and environmental impact across the material lifecycle–considerations that have become increasingly relevant given recent progress in CO_2_‐derived polymers, bio‐based materials, and green chemistry approaches.^[^ ^21^ ^]^

Here, the intersection of SICPs with sustainable chemistry is considered. First, synthesis strategies, including renewable feedstocks, CO_2_‐based polymers, and green polymerization methods, are highlighted. Second, applications of SICPs in lithium‐based batteries are explored, focusing on the consequences of their implementation as electrolytes, binders, and interface components to improve battery performance efficiency and recycling prospects. SICPs have been developed for various types of batteries beyond Li‐ion, including more earth‐abundant sodium, potassium, and zinc systems.^[^ ^22^ ^]^ They also play roles in fuel cells and supercapacitors, where nanostructured architectures and ion‐selective properties advance energy conversion efficiency.^[^ ^4^ ^]^ Throughout, we emphasize approaches that could enable commercial sustainable materials while maintaining the ionic conductivity and stability required for practical implementation.

## 2 Commercial Viability and Promising Sustainable Synthetic Routes

**Figure 2:**
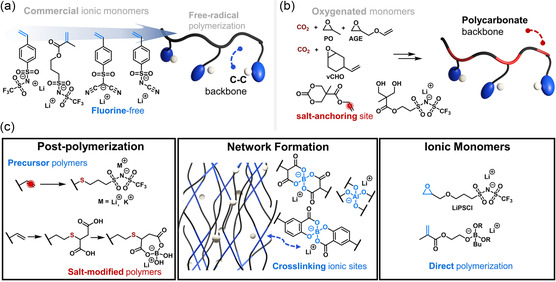
a) Scalable vinyl monomers commercially available for C—C backbone SICP synthesis. b) Alternative approach to potentially sustainable polycarbonate backbones utilizing CO_2_/epoxide ROCOP and cyclic carbonate ROP, followed by postpolymerization ionic functionalization, alongside polycondensation of salt‐modified diols with dimethyl carbonate.[^34^, ^37^] c) Effective salt introduction strategies: thiol‐ene postpolymerization functionalization,^[^ ^39^ ^]^ ionic crosslinking^[^ ^41^ ^]^ and direct polymerization of salt‐based monomers.[^37^, ^44^]

SICPs design requires careful consideration of multiple structural elements that collectively determine their performance in energy storage applications. Polymer backbone and side‐chain chemistry control the glass transition temperature (*T*_g_) and local chain dynamics, which directly impact ion transport mechanisms. High dielectric constants, achieved through strategic incorporation of heteroatoms, promote the crucial dissociation of tethered anions from their Li‐counterions.^[^ ^23^ ^]^ Among the various anionic groups, sulfonamides and borates have emerged as particularly effective choices for Li‐ion transport.^[^ ^24^ ^]^ Conventional dual‐ion conducting systems rely on relatively costly lithium salts such as LiTFSI. In contrast, SICPs offer advantages as single‐component systems with ionic moieties already incorporated, though scalable synthetic routes remain crucial for commercial viability.^[^ ^25^ ^]^ Recent advances have demonstrated their industrial potential through successful continuous processing methods suitable for large‐scale manufacturing.^[^ ^10^ ^]^ A milestone in SICP commercialization came in 2017 when specific polymers introduced two ionic monomers based on methacrylate and styrene (**Figure** **2**a).^[^ ^26^ ^]^ The commercial landscape has expanded, with other companies such as Polykey polymers now offering single‐ion conducting polymers for battery applications.^[^ ^27^ ^]^

Initial commercial materials featured the trifluoromethane sulfonimide (TFSI) anion, showing particular effectiveness in block copolymers with poly(ethylene oxide) (PEO)^[18b,c]^ and, more recently, in composites with nanostructured inorganics.^[^ ^28^ ^]^ However, sustainability concerns surrounding fluorinated components have driven recent innovations,^[^ ^29^ ^]^ culminating in the commercial development of nonfluorinated alternatives capable of hundred‐gram batch production (Figure 2a).^[^ ^26^ ^]^ These fluorine‐free SICPs, based on (styrenesulfonyl)(dicyano)methide salt and lithium poly(vinyl alcohol oxalate borate), offer benefits beyond environmental considerations.^[^ ^26^, ^27^ ^]^ The dicyanomethide systems exhibit higher thermal stability (383 vs. 358 °C) than TFSI‐based counterparts,^[^ ^30^ ^]^ while the borate‐based polymers form protective layers between battery components that stabilize high‐voltage cathodes and reactive metal anodes.^[^ ^31^ ^]^

Polymerization of vinyl‐containing ionic monomers leads to purely C—C backbones, while alternative polycarbonate backbones offer enhanced potential for chemical recycling and sustainable synthesis through renewable resource use (Figure 2b).^[^ ^32^ ^]^ A promising approach involves the ring‐opening copolymerization (ROCOP) of CO_2_ with epoxides. This method has leveraged propylene oxide (PO), produced at multimillion‐ton scale annually,^[^ ^33^ ^]^ and allyl glycidyl ether (AGE).^[34a]^ Using a zinc glutarate catalyst, these monomers yield amorphous low *T*_g_ polycarbonates with pendant allyl groups suitable for postpolymerization functionalization via thiol‐ene chemistry to introduce ionic groups. Significant progress in ROCOP catalysis beyond zinc glutarate provides high polycarbonate selectivity and molar mass control while operating under milder conditions of temperature and CO_2_ pressure (<10 bar).^[^ ^35^ ^]^ Biorenewable feedstocks are increasingly viable, with epichlorohydrin derived from glycerol (a biodiesel industry byproduct) and sugar‐based monomers offering sustainable pathways to SICPs.^[^ ^36^ ^]^ Recently, 4‐vinyl cyclohexene oxide (vCHO)/CO_2_ ROCOP alongside cyclic carbonate ring‐opening polymerization (ROP) yielded polycarbonate SICPs with subsequent functionalization to lithium borate groups for enhanced ion‐pair dissociation.^[37a]^ Direct incorporation of ionic functionality is also achievable through polycondensation using lithium sulfonamide‐modified diols (with methanol/water elimination) or through more atom‐economic polyaddition chemistry to form polyurethanes.[^34^, ^38^] ROP and ROCOP approaches operate at more moderate temperatures (25–80 °C) and maximize atom economy (no small molecule elimination), though direct polymerization of anionic monomers by these methods requires careful consideration due to potential catalyst interference and undesired chain initiation.

The development of efficient polymer postfunctionalization reactions has proven particularly valuable for reducing the complexity of SICP synthesis, enabling control over ionic group content while maintaining backbone integrity. Modern approaches to salt introduction predominantly leverage thiol‐ene coupling (Figure 2c), which typically achieves over 95% conversion under mild conditions of room temperature and UV irradiation.^[^ ^39^, ^40^ ^]^ While TFSI‐based thiol approaches require multistep syntheses that could limit scalability, they provide excellent control over ionic functionality. Complementary lithium borate strategies offer more straightforward synthetic routes utilizing dicarboxylic acids and abundant precursors such as boric acid and lithium carbonate.[^37^, ^41^] The synthetic toolkit has further expanded through alternative functionalization methods, from Mitsunobu reactions to substitute hydroxyl groups on polyether backbones^[^ ^42^ ^]^ or ionization through nucleophilic substitution of pendant chlorides.^[^ ^43^ ^]^ Network formation represents another simplified approach to SICP synthesis, where highly processable oligomers can be crosslinked through their hydroxyl end‐groups to form borate, aluminate, and silicate single‐ion conductors.^[41a,d]^ Recent work has demonstrated the effective use of thiol‐ene networking reactions with vinyl lithium borate precursors derived from biorenewable dicarboxylates.^[41b,c]^ Direct polymerization routes using lithium sulfonamide‐modified epoxides offer additional synthetic possibilities, though questions remain about the optimal stage for ionic group introduction.^[^ ^44^ ^]^ Among sustainable manufacturing routes, water‐based emulsion polymerizations yield well‐defined SICP nanoparticles.^[^ ^45^ ^]^ These particles are effective enhancers of conductivity and interfacial stability in electrolytes and electrodes.

**Figure 3:**
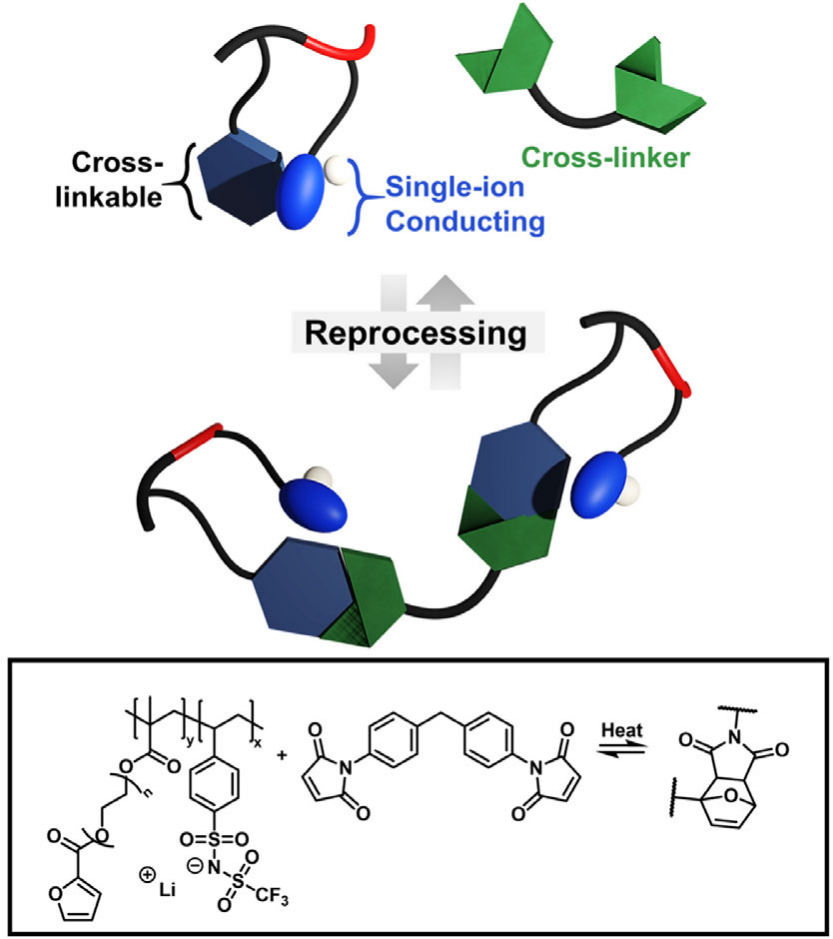
Reprocessing and recycling of SICPs utilizing reversible dynamic covalent chemistry, which has been validated for furan‐maleimide Diels–Alder/retro‐Diels–Alder equilibrium.^[^ ^46^ ^]^

Advancing sustainable end‐of‐life strategies for polymeric materials requires careful consideration of SICP recyclability and recovery pathways. Furan‐maleimide dynamic covalent chemistry demonstrates successful SICP reprocessing under moderate conditions (140 °C) with retained ionic conductivity (**Figure** **3**).^[^ ^46^ ^]^ These thermo‐reversible bonds enable multiple reprocessing cycles while maintaining structural stability under normal operating conditions. Other reversible chemistries, including boronic ester exchange and spiroborate linkages should also be promising for next‐generation reprocessable SICPs.^[^ ^47^ ^]^ However, in battery applications, side‐reactions may lead to accumulation of by‐products that limit reprocessing cycles. While conventional polycarbonates, polyethers, and polyesters can achieve high monomer recovery (>90%) through chemical recycling at moderate temperatures (<100 °C), the effectiveness of these approaches for polymers containing ionic groups remains unexplored.^[^ ^32^ ^]^ The selective (bio)degradation of ester and carbonate linkages in the presence of ionic functionalities presents new opportunities for investigation, with both polymer recycling and salt recovery strategies advancing the potential for circular materials design.^[^ ^48^ ^]^

## 3 Advancing Lithium Battery Performance

The transition to sustainable energy storage has positioned lithium metal batteries (LMBs) as a critical technology, offering theoretical capacities approaching nearly ten times that of conventional graphite anodes and enabling cell‐level energy densities of ≈450 Wh kg^−1^.^[^ ^49^ ^]^ This substantial increase addresses electric vehicle adoption barriers by enabling extended driving ranges with reduced battery pack weights.^[^ ^49^ ^]^ However, the commercial viability of LMBs has been historically constrained by safety concerns related to challenges in lithium metal stabilization and poor cycling performance due to uncontrolled lithium deposition and dendrite formation (lithium filaments).^[^ ^49^ ^]^

**Figure 4:**
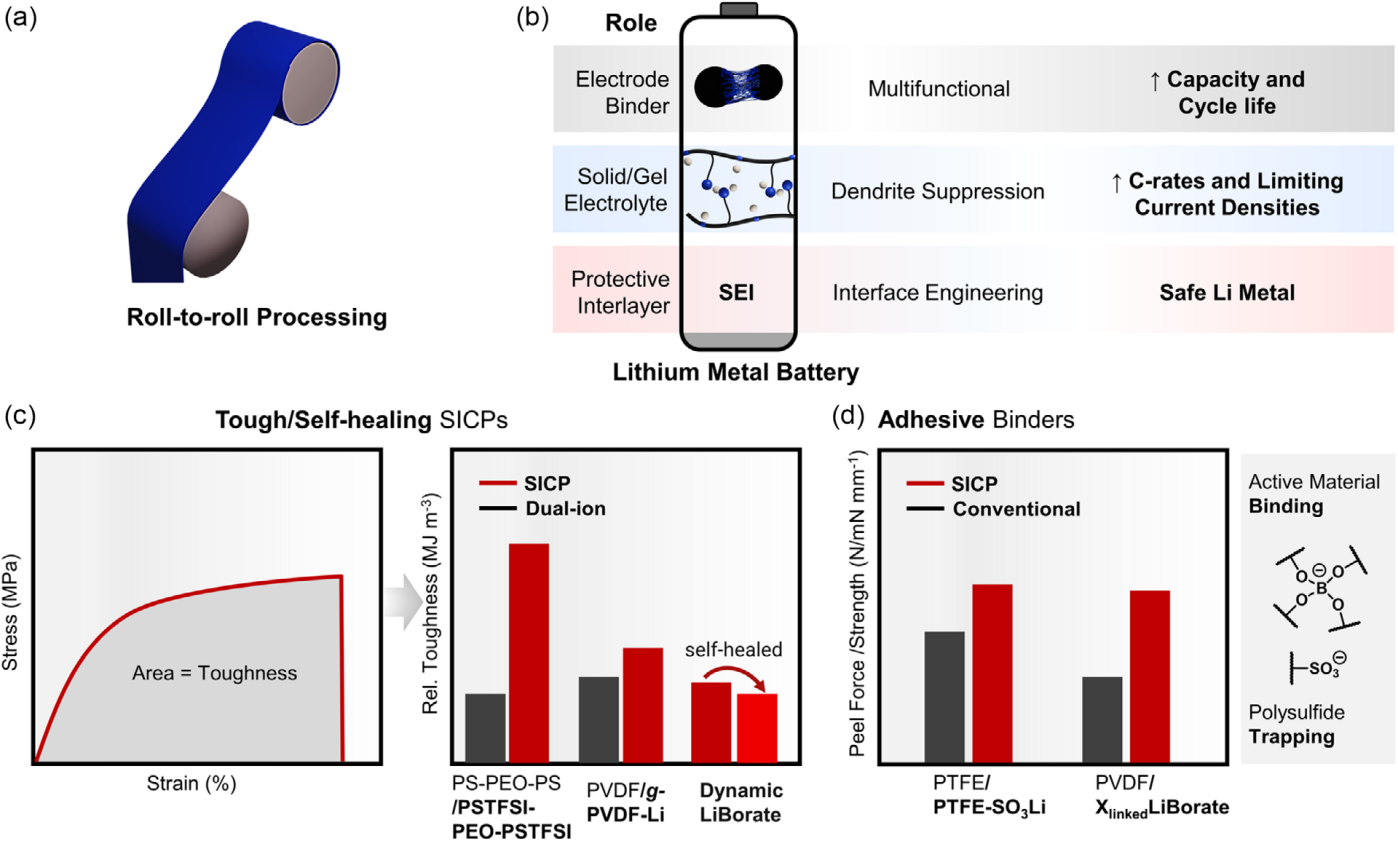
a) Processability of SICPs into thin films, highlighting their potential for scalable manufacturing. b) Key roles in battery energy storage, including the formation of SEIs. c) Enhanced mechanical properties of SICPs compared to dual‐ion conductors, with relative toughness values derived from stress–strain curves.[^18^, ^61^] d) Adhesive performance of SICPs, demonstrated through nanoindentation^[^ ^60^ ^]^ and peel tests^[^ ^62^ ^]^ (relative values given). These properties contribute to the sustainability of SICPs by improving battery performance and enabling solvent‐free/aqueous electrode processing and recycling practices.

Current electrolyte technologies present distinct trade‐offs. Liquid electrolytes, while offering superior bulk conductivity (≈10–100 mS cm^−1^) are flammable and prone to concentration polarization effects that promote dendrite formation.^[^ ^50^ ^]^ Traditional polymer electrolytes (<1 mS cm^−1^) improve safety but suffer from limited ion selectivity (*t*_+_ 0.2–0.5) and long‐term stability against lithium metal.^[^ ^2^ ^]^ Ceramic electrolytes, from brittle garnet‐oxides to more compliant sulfide‐argyrodite systems, achieve selective ion transport (*t*_+_ ≈ 1) with high conductivities (1–10 mS cm^−1^) but struggle with interfacial stability, particularly as mechanical mismatches can lead to contact loss during cycling.^[^ ^51^ ^]^ SICPs offer a pathway to balancing these features, combining ion selectivity with mechanical flexibility. Their immobilized anions address concentration gradients while maintaining the ability to be processed into thin films (<20 μm) at scale, reducing overall battery weight and volume.^[^ ^52^ ^]^ Crucially, recent work demonstrated roll‐to‐roll processable SICPs fabricated into industrially relevant cylindrical and Z‐stack pouch cells (**Figure** **4**).^[^ ^10^ ^]^

SICPs are often reported for their success in dendrite suppression.[^31^, ^53^] Recently, network SICPs incorporating immobilized–SO_3_ groups highlighted this capability, achieving stable cycling in Li‐symmetric cells for over 2200 h (at 0.2 mA cm^−2^) while comparable dual‐ion polymer systems degraded after just 580 h.^[53a]^ The network architecture demonstrated superior performance metrics, achieving *t*_+_ of 0.92 (compared to 0.22 for dual‐ion systems) and ionic conductivity of 1.1 mS cm^−1^ (vs 0.23 mS cm^−1^). These benefits translated to full‐cell performances, with network SICP‐cells demonstrating better rate capability, delivering 145 mAh g^−1^ with commercial LiFePO_4_ cathodes at 0.1C‐rate and maintaining 64 mAh g^−1^ at 6C, while dual‐ion systems failed above 1C.

This stability stems from multiple factors, including the formation of a more uniform solid electrolyte interphase (SEI) due to the absence of mobile anions. Mechanistic studies of boron‐containing SICPs have revealed that fixed anions prevent anion aggregation at the lithium anode, leading to more uniform electric field distributions and suppressed dendrite growth.^[31a]^ The enhanced interfacial stability sustained cycling performance in Li‐symmetric cells for over 5400 h at 0.25 mA cm^−2^, whereas cells without the SICP shorted at the 315th hour. Through the formation of a B‐containing stable interlayer and reduced parasitic reactions, Li metal Li_1.2_Ni_0.2_Mn_0.6_O_2_ cathode cells achieved 245 mAh g^−1^ at high voltages (up to 4.8 V).

The dendrite resistance of SICPs is enhanced by their distinctive mechanical properties through ionic crosslinking mechanisms.^[18b]^ While high shear moduli (*G*′) ceramic electrolytes (>10 GPa) can initially block dendrite initiation, they fail once dendrites nucleate at high current densities (1–2 mA cm^−2^).^[^ ^54^ ^]^ SICPs combine moderate *G*′ (≈100 kPa) with sophisticated toughening and viscoelastic properties.^[^ ^55^ ^]^ Strategic ionic functionalization enhances mechanical toughness^[^ ^56^ ^]^ while dynamic ionic crosslinking enables self‐healing capabilities that potentially extend battery lifetimes (Figure 4c).^[31b]^ Notably, some SICP‐electrolytes have achieved current densities up to ≈2.5 mA cm^−2^ at 25 °C, surpassing liquid electrolytes’ typical range of 0.5–1.5 mA cm^−2^ under comparable conditions.[^20^, ^53^, ^54^]

Complementing these stability mechanisms and mechanical advantages, interface engineering has advanced through in situ formed interfaces where precursor molecules polymerize directly at the lithium metal surface.[^41^, ^57^] These interfaces function as standalone electrolytes and protective interlayers for other electrolyte systems.^[^ ^58^ ^]^ The culmination of these developments was exemplified in recent work with SIC‐based multiblock co‐poly(arylene ether sulfone)s.^[^ ^59^ ^]^ These systems, incorporating propylene carbonate plasticizer and designed for reduced fluorine content, enabled high‐energy lithium‐metal batteries with industrial‐scale LiNi_0.8_Mn_0.1_Co_0.1_O_2_ (NMC811) cathode loadings (10.6 mg cm^−2^). They reported stable performance over 500 cycles at 5C without significant capacity loss.

Beyond their role as electrolytes, SICPs serve as valuable multifunctional electrode binders whose tunable chemistry enables optimization across diverse battery chemistries. Traditional poly(vinylidene fluoride) (PVDF) binders, while providing basic mechanical adhesion through van der Waals interactions, lack the enhanced interfacial bonding achieved through SICP ionic and hydrogen‐bonding interactions (Figure 4d).^[^ ^60^ ^]^ The superior adhesion arises from fixed negative charges forming robust interfaces with active materials and current collectors, maintaining electrode integrity during cycling‐induced volume changes.^[37a]^ This enhanced stability has proven effective in high‐voltage systems: lithium‐sulfonated variants of PVDF^[^ ^61^ ^]^ and PTFE^[^ ^60^ ^]^ show improved interfacial contact and cyclability compared to conventional PVDF/PTFE binders. Borate‐based SICPs combine high ionic conductivities (>10^−4^ S cm^−1^) with extensive hydrogen‐bonding networks to improve capacity and capacity retention in NMC811 cathodes.^[37a]^ The adaptable chemistry particularly benefits next‐generation lithium–sulfur batteries, where boron coordination sites can address polysulfide shuttling while leveraging sulfur's natural abundance.^[^ ^62^, ^63^ ^]^ The sustainability benefits extend to manufacturing processes, as SICPs enable aqueous and dry‐processing alternatives to the toxic *N*‐Methyl‐2‐pyrrolidone (NMP) solvents required for PVDF electrode fabrication and improve recovery potential.^[^ ^60^ ^]^ This aligns with UN sustainability goals, particularly significant given projections for the global battery binders market to reach $7.5 billion by 2032 (CAGR 7.9%).^[^ ^49^, ^64^ ^]^

## 4 Diversifying SICP Applications for Alternative Energy Storage

SICPs extend beyond lithium systems into emerging battery technologies using earth‐abundant elements. In sodium–metal batteries, two recent approaches demonstrate this adaptability: multiblock poly(arylene ether sulfone) electrolytes with tethered TFSI anions achieve 2.6 mS cm^−1^ conductivity at 40 °C and 0.96 sodium transference numbers,^[^ ^65^ ^]^ while polymerized 2‐acrylamido‐2‐methylpropanesulfonic acid sodium salt (AMPSNa) creates specialized sodium channels (0.43 mS cm^−1^, *t*_+_ 0.82).^[^ ^66^ ^]^ Both systems form protective Na_2_O/Na_3_N/NaF surface layers, enabling stable cycling. The multiblock system maintains 85% capacity over 1000 cycles at 0.2C with Na_3_V_2_(PO_4_)_3_ cathodes, while the AMPSNa system's weaker binding energy (−5.20 vs −9.11 eV for NaTFSI) enables fast 4C charging even at low 0 °C temperatures. These surpass conventional PVDF‐HFP/NaTFSI electrolytes’ conductivity (≈10^−6^ S cm^−1^) and transference (0.2–0.4), which suffer from unstable interfaces containing decomposed TFSI anions and Na_2_CO_3_.

For aqueous zinc batteries, hydrogel SICP electrolytes are a promising platform for divalent ion transport.^[22d,e]^ Pseudo‐polyrotaxane structures combining PEO chains with α‐cyclodextrin rings achieve conductivities of 22.4 mS cm^−1^ and Zn^2+^ transference numbers of 0.92 at 60% water content.^[22e]^ In this example, the cyclodextrin molecules form confined channels for zinc‐ion transport, preventing the uneven metal deposition that plagues conventional systems. Where dual‐ion electrolytes develop significant dendrites within 40 cycles, these hydrogels enable stable zinc plating for 160 h at 1 mA cm^−2^ and >90% capacity retention over 3000 cycles in full cells. Alternative sulfonate‐based frameworks achieve similar ion selectivity (*t*_+_ 0.91) but lower conductivity (0.22 mS cm^−1^), highlighting the ongoing challenge of balancing ion selectivity with transport kinetics. These hydrogel systems show particular promise for flexible electronics applications.^[22e]^

Another direction combines single‐ion conduction with redox‐active moieties in copolymer architectures, creating multifunctional materials that simultaneously facilitate ion transport and participate in charge storage.^[^ ^67^ ^]^ While many of these advances rely on petroleum‐based precursors, research continues into sustainable alternatives.^[^ ^68^ ^]^ These developments in SICP technology support the advancement of grid‐scale energy storage, where their performance characteristics align with the requirements for renewable energy integration.

## 5 Summary and Outlook

The development of SICPs has progressed across multiple fronts, from synthesis to practical battery implementation. The development aligns with UN Sustainable Development Goals, particularly in advancing clean energy storage (SDG 7) and responsible materials production (SDG 12). Commercial availability of ionic monomers, efficient thiol‐ene chemistry, and networking approaches demonstrates promising production routes, while CO_2_‐based synthesis and use of renewable feedstocks offer paths to greener manufacturing. Dynamic covalent chemistries confer the potential for enabling circular material flows. In lithium–metal batteries, SICPs show promising dendrite mitigation through combined mechanical and ionic mechanisms, though optimization of liquid additives remains ongoing. Implementing water‐based and solvent‐free electrode fabrication with inherently adhesive SICP binders demonstrates potential for reducing environmental impact across manufacturing, though comprehensive life cycle assessment studies remain needed to quantify these benefits. Progress toward industrial‐scale NMC811 cathode loadings and scalable continuous processing methods, alongside applications in sodium and zinc battery systems, strengthens the sustainability case for these materials.

Looking ahead, commercial implementation requires balancing ionic conductivity with mechanical robustness while scaling up synthesis methods and establishing cost‐effective recycling systems with quantitative sustainability metrics. An intriguing concept is the potential use of SICPs as pressure‐sensitive adhesives for pouch cell assembly, which could facilitate more efficient battery disassembly and material recovery than traditional permanent adhesives. Success in commercialization demands careful balance of performance requirements with environmental benefits across the technology lifecycle. The demonstrated versatility of SICPs in both high‐performance and earth‐abundant battery systems positions them as valuable contributors to sustainable energy storage, provided these key challenges can be effectively addressed through continued research and development.

## Conflict of Interest

The authors declare no conflict of interest.
